# Unmet Needs and Caregiving Burden Among Informal Caregivers of Older Adults: A Qualitative Study

**DOI:** 10.3390/nursrep16100358

**Published:** 2026-10-10

**Authors:** Aliya Zhylkybekova, Gulbakit K. Koshmaganbetova, Kulman Nyssanbayeva, Bayan Nurgaliyeva, Gulbanu Mukishova, Azhar N. Zhexenova, Lida Aliyeva, Nurgul Ablakimova, Andrej M. Grjibovski, Natalya Glushkova

**Affiliations:** 1 Department of Pathological Physiology, West Kazakhstan Marat Ospanov Medical University, 030000 Aktobe, Kazakhstan; 2 Department of Evidence-Based Medicine and Scientific Management, West Kazakhstan Marat Ospanov Medical University, 030000 Aktobe, Kazakhstan; 3 Department of Propaedeutic of Internal Diseases, Asfendiyarov Kazakh National Medical University, 050000 Almaty, Kazakhstan; 4 Department of Pharmacology, West Kazakhstan Marat Ospanov Medical University, 030000 Aktobe, Kazakhstan; 5 Rectorate, Reaviz University, 198095 Saint Petersburg, Russia; 6 Department of Epidemiology and Modern Vaccination Technologies, I.M. Sechenov First Moscow State Medical University, 105064 Moscow, Russia; 7 Department of Healthcare Organization and Preventive Medicine, North-Eastern Federal University, 677000 Yakutsk, Russia; 8 Department of Health Policy and Management, Al-Farabi Kazakh National University, 050000 Almaty, Kazakhstan; 9 Health Research Institute, Al-Farabi Kazakh National University, 050000 Almaty, Kazakhstan

**Keywords:** informal caregivers, older adults, caregiving burden, social support, qualitative study, Kazakhstan

## Abstract

**Background:** Informal caregivers play a central role in supporting older adults with functional limitations, yet caregiving may impose substantial physical, psychological, social, and occupational demands. Objectives: This study aimed to explore caregiving experiences, sources of burden, and unmet healthcare and social support needs among informal caregivers of older adults with high caregiver burden. **Methods:** This qualitative study represented the second phase of a sequential explanatory mixed-methods study. Ten caregivers were purposively selected from the preceding quantitative phase based on a high caregiver burden, Caregiver Strain Index Plus (CSI+) score ≥ 7. In-depth semi-structured interviews were conducted in Kazakh or Russian across four cities of Kazakhstan and analyzed using inductive reflexive thematic analysis. **Results:** Six themes were identified: (1) physical and psychological impact of caregiving; (2) work–care balance and social isolation; (3) gender roles in the care of older adults; (4) insufficient support from healthcare and social services; (5) unmet caregiver needs; and (6) limited awareness of available support and services. Participants described increasing physical and emotional strain, disruption of employment and social life, and gendered expectations surrounding family caregiving. They also reported limited professional guidance and recognition, unmet needs for respite and practical assistance, and limited awareness of available health and social services, with informal networks and online sources often used to obtain information. **Conclusions:** Strengthening caregiver support within primary healthcare and improving access to information, practical assistance, and respite services may help address the needs identified among caregivers with high caregiver burden.

## 1. Introduction

The World Health Organization (WHO) estimates that by 2030, the number of people aged 60 and older will rise to 1.4 billion, up from 1 billion in 2020. By 2050, this figure is expected to double to 2.1 billion. Over the same period, the population aged 80 and above is projected to triple, reaching 426 million [1]. These demographic transitions are equally pronounced in the Republic of Kazakhstan. Projections from the United Nations Population Fund indicate that by 2050, older adults will comprise approximately 14% of the national population, nearly doubling from 7.5% in 2019 [2]. A sociological study conducted in 2020 further demonstrated the predominance of family-based care, showing that 64% of older adults resided in multigenerational households. When physical assistance was needed, 69% relied primarily on their children, while only 0.8% accessed formal social services [3].

The growing number of older adults is accompanied by an increase in chronic diseases and a rising need for comprehensive care, which in turn places additional pressure on healthcare systems [4,5]. To mitigate the strain on healthcare and social services, governments increasingly rely on family members to provide care for older adults. Informal caregiving is often viewed as a cost-effective alternative to institutionalization, as it reduces older adults’ use of healthcare facilities and thereby lowers expenditures on medical services [6]. At the same time, this perspective tends to overlook the health and well-being of caregivers themselves, despite their indispensable contribution to sustaining care provision. Caregivers should be regarded as “invisible second patients” [7], as they also require professional support from healthcare and social services, just as the care recipients themselves. Yet, healthcare professionals often adopt a patient-centered approach that focuses solely on the individual receiving care, leaving the needs of family caregivers largely unaddressed [8].

Caregivers have diverse support needs that extend beyond the medical care provided to care recipients. These include access to formal health and social care services, clear information about the care recipient’s condition and available services, practical guidance on caregiving, and psychological and emotional support [9]. However, caregivers often report difficulties obtaining relevant information, uncertainty about where to seek assistance, and a discrepancy between the support they expect and the services actually available [10]. Limited recognition of caregivers’ needs by healthcare and social service providers may further exacerbate these challenges. Educational and supportive interventions may help caregivers develop caregiving skills, manage stress and anxiety, and reduce the risk of burnout [11]. These needs are particularly important given that caregiving can extend beyond health-related impacts, often disrupting employment and career opportunities [12], while also adversely affecting caregivers’ physical and psychological well-being and their ability to participate in the labor force [13].

Caregiver burden was conceptualized by Zarit and colleagues (1980) as the extent to which caregiving responsibilities affect caregivers’ emotional and physical health, social life, and financial well-being [14]. The magnitude of this burden is shaped by multiple factors. Higher levels of burden have been reported among women [15,16] and among caregivers providing more intensive assistance with activities of daily living (ADL) and instrumental activities of daily living (IADL) [17]. The Stress Process Model proposed by Pearlin and colleagues provides a broader framework for understanding these experiences by conceptualizing caregiving burden as arising from the interplay of caregiving-related stressors, secondary strains affecting other areas of life, and the personal and social resources available to caregivers [18].

In Kazakhstan, support for older adults with care needs is provided through both primary healthcare and the social welfare system. Primary healthcare teams include physicians, nurses, social workers, and psychologists, with social workers responsible for assessing social needs, providing guidance to family members on home-based care, and facilitating access to health and social services. Special social services, including home-based support, are also available to eligible older adults and persons with disabilities who meet established criteria. However, these arrangements are primarily structured around the needs and eligibility of the care recipient rather than the systematic identification and assessment of informal caregivers as individuals with their own support needs [19,20,21,22]. The everyday experiences of informal caregivers of older adults and the broader circumstances contributing to their burden remain insufficiently understood. In particular, little is known about the caregiving experiences, sources of burden, and unmet healthcare and social support needs of informal caregivers of older adults with high caregiver burden in Kazakhstan.

## 2. Materials and Methods

### 2.1. Study Design

This qualitative descriptive study represents the second, qualitative phase of a sequential explanatory mixed-methods study among informal caregivers of older adults in Kazakhstan, with the overall study design and procedures detailed in a previously published protocol [23]. The first, quantitative phase was conducted in Astana, Almaty, Semey, and Aktobe and assessed caregiver burden, quality of life, health-related quality of life, and factors associated with informal caregiving among caregivers of older adults. The findings showed that 15.3% of caregivers met the criterion for high caregiver burden Caregiver Strain Index plus (CSI+) score ≥ 7 [16]. Caregivers with high burden were subsequently purposively selected for the qualitative phase to explore their caregiving experiences and sources of burden in greater depth.

The qualitative phase was conducted at the same four sites as the preceding quantitative phase. These cities served as recruitment locations rather than separate analytical settings, as the study was not designed to compare caregiving experiences geographically. Accordingly, all interviews were analyzed as a single dataset.

This study was reported in accordance with the Consolidated Criteria for Reporting Qualitative Research (COREQ) guidelines [24]. The completed COREQ checklist is provided as Supplementary File.

### 2.2. Research Team and Reflexivity

All interviews were conducted by the first author, PhD, a female researcher affiliated with West Kazakhstan Marat Ospanov Medical University, Kazakhstan. She had formal training and previous experience in qualitative research methods, including qualitative interviewing and thematic analysis. The interviewer’s interest in the topic arose from her previous research on informal caregiving and caregiver burden in Kazakhstan. Given her involvement in the quantitative phase of the study, the first author was aware that her prior knowledge of participants’ burden scores and the quantitative findings could influence the emphasis placed on particular issues during interviewing, coding, and interpretation. To address this potential influence, reflexive field notes were maintained throughout data collection and analysis, and emerging interpretations were discussed with the research team to encourage consideration of alternative interpretations and to keep the analysis grounded in participants’ accounts.

### 2.3. Setting, Participants, and Recruitment

Caregivers were eligible if they were aged 18 years or older, had a high caregiver burden (CSI+ score ≥ 7) in the preceding quantitative phase, provided informal care to an older adult aged 65 years or older with at least two limitations in activities of daily living (ADL), had been providing care for at least two weeks, were proficient in Kazakh or Russian, and provided informed consent. No further selection criteria were applied based on recruitment site, relationship to the care recipient, or other caregiving circumstances. The interval between the CSI+ assessment in the quantitative phase and the qualitative interview was approximately 6–12 months. Caregiver burden and caregiving circumstances were not reassessed before the qualitative interview.

Fourteen caregivers were approached, of whom four declined participation and ten were interviewed. Of the ten participants, seven were recruited in Aktobe and one each in Astana, Almaty, and Semey. Three interviews were conducted in Kazakh and seven in Russian. The CSI+ consists of 18 items, including 13 items assessing negative aspects of caregiving and five items assessing positive aspects. The total score ranges from −5 to 13, with higher scores indicating greater caregiver burden [25].

### 2.4. Data Collection

A semi-structured interview guide was developed based on a review of the literature on informal caregiving for older adults [26]. The guide was pilot tested with three family caregivers, whose interviews were not included in the final analysis, and minor wording adjustments were made for clarity. The final semi-structured interview guide, including the main questions and principal probes, is provided in Supplementary Table S1.

Data were collected between December 2023 and May 2024. Interviews were conducted in person at a time and location convenient for participants, either in a private room at a healthcare facility or at the participant’s home, according to their preference. Interviews lasted approximately 30–45 min, were audio-recorded, and were supplemented with field notes documenting contextual information and non-verbal cues. No relationship existed between the interviewer and participants before recruitment. Before each interview, participants were informed about the researcher’s professional background, her role in the study, and the purpose of the research. Written informed consent was obtained. Interviews were conducted in Kazakh or Russian according to participants’ language preferences.

### 2.5. Data Analysis

All interview transcripts were analyzed using inductive reflexive thematic analysis following Braun and Clarke’s six-phase approach [27]. The published study protocol initially specified inductive content analysis [23]. However, the decision to use reflexive thematic analysis was made following completion of data collection and before formal coding and theme development began. This approach was considered better suited to identifying and interpreting patterns of shared meaning across caregivers’ accounts. Consistent with this revised analytic approach, saturation was not used as a formal criterion for determining sample adequacy. The decision to focus the qualitative phase on caregivers with high burden (CSI+ score ≥ 7) was introduced after the quantitative phase and was not specified as an eligibility criterion in the published protocol. This refinement was made to strengthen the explanatory connection between the quantitative and qualitative phases and to enable an in-depth exploration of the experiences and circumstances associated with high caregiver burden.

The analysis began with repeated reading of the transcripts for familiarization and initial observations. Relevant data segments were systematically coded by the first author, and related codes were examined to develop candidate subthemes and themes. The thematic structure was iteratively reviewed and refined for coherence within themes, meaningful distinctions between themes, and relevance to the research questions. Developing codes, subthemes, and themes were discussed with the research team during regular analytical meetings, with reference to the original transcripts. Coding and theme development were inductive and were not guided by the Stress Process Model or other pre-existing theoretical frameworks. The final thematic structure, including themes, subthemes, and illustrative codes, is presented in Supplementary Table S2.

After the ten interviews had been completed, the research team considered the adequacy of the dataset in relation to the study aim, the depth and relevance of participants’ accounts. The dataset was considered sufficiently information-rich to develop and interpret coherent patterns of shared meaning across the six themes.

## 3. Results

### 3.1. Sociodemographic Characteristics of Caregivers

Participants were predominantly women (*n* = 9, 90%) with a mean age of 40.9 years (SD = 10.8; range 28–58). Most respondents were married (60%) and held higher education (90%). Care was primarily provided for parents (50%), followed by spouse’s parents (30%) and grandparents (20%). These percentages refer to caregivers rather than care recipients. Two participants cared for more than one relative, and all care recipients met the study eligibility criteria. For these participants, the relationship category and caregiving duration reported in Table 1 refer to the primary care recipient. In terms of employment, 40% were unemployed and 60% were employed. Dementia or memory impairment was the most frequently reported condition (40%), followed by post-stroke status (20%). Demographic characteristics are summarized in Table 1.

The thematic analysis identified six themes: (1) physical and psychological impact of caregiving; (2) work–care balance and social isolation; (3) gender roles in the care of older adults; (4) insufficient support from healthcare and social services; (5) unmet caregiver needs; and (6) limited awareness of available support and services. The first three themes reflected the personal, social, and family dimensions of caregiving, whereas the final three concerned distinct but interrelated aspects of caregiver support. Insufficient support concerned limitations in the professional response of healthcare and social services, unmet needs reflected the forms of assistance caregivers reported needing, and limited awareness concerned caregivers’ knowledge of and ability to navigate available services.

### 3.2. Physical and Psychological Impact of Caregiving on Caregivers

Participants described both physical and psychological impacts associated with caregiving. In their retrospective accounts, many participants described caregiving as becoming more demanding as the care recipient’s illness progressed. This was especially evident in tasks requiring physical assistance, such as hygiene procedures and mobility support, which participants described as becoming increasingly difficult over time.

“I take care of her as if she were a small child. I change her diapers, wash her, dry her, put on a new diaper, change her socks, wash her face. She cannot walk or move on her own. At first, I lifted her myself, but later we had to do it together; we take her to the toilet and the bathroom. It became too difficult for me to lift her alone. Only the two of us can manage.”

(Informant 1)

“It is very difficult. Especially when she became bedridden and could no longer get up. It was hard to move her, to lift her, and to bathe her.”

(Informant 5)

“I need psychological support. I want to see a psychologist because lately I often lose my temper. I lack communication, I stay at home and I miss new information. I want to learn something new, but I cannot, I have no time.”

(Informant 2)

Particularly intense psychological strain was described by caregivers of older adults with dementia. For example, a daughter caring for her mother with dementia described her condition in response to her mother’s needs:

“I sometimes think I might die myself, because I don’t sleep at night [my mother is restless and does not sleep at night], and I need to get some rest. I wonder how much longer I can endure this. I say to myself: ‘Lord, why does old age have to be like this?”

(Informant 4)

A granddaughter caring for her grandmother with dementia similarly described the emotional impact of behavioral changes and nighttime disturbances:

“My grandmother, my grandmother, my beloved grandmother ‘died’ immediately after the diagnosis. After that, the person who is with us is just a shell. Nighttime is her favorite time, and the wardrobe is her favorite place. She would spend the whole night searching for clothes in that wardrobe. We couldn’t sleep at night. The light is on, she is looking for something and scolding us.”

(Informant 10)

In addition, some participants were caring for multiple care recipients, which led to even greater difficulties.

“In the last six months, we also had an uncle who was ill, but he passed away. It may sound wrong to say, but I will say it anyway. In some way, it made my life a little easier. Because two people are not the same as one. And caring for two people is, of course, much harder—it takes more time and carries greater responsibility.”

(Informant 6)

### 3.3. Work–Care Balance and Social Isolation

Participants described caregiving as affecting both their ability to maintain employment and their participation in social life. Some had left the workforce or adjusted their work arrangements because of caregiving responsibilities, while limited opportunities to leave the care recipient unattended restricted their social activities and contact with others.

“We are literally with [my mother] around the clock. My sister takes care of her during the first half of the day, and I take over in the second half. I work full time. My sister spoke with her management, and half of her work she does online.”

(Informant 8)

“Even if I can take time off, this cannot go on endlessly, I cannot make another person do my work. I cannot force someone else to perform my duties, especially if they are not being paid for it.”

(Informant 6)

One of the study participants, who was caring for her mother, had to quit her job and leave her husband, moving to another city in order to provide care.

“I had to move [to another city]. I left my job and my husband in order to take care of my mother.”

(Informant 4)

In addition, participants highlighted social isolation caused by the inability to leave older adults unattended. Some study participants reported that for extended periods— sometimes up to 15 days—they did not leave their homes due to the lack of assistance from others. These circumstances created significant barriers to their social interaction and participation in community life.

“If I need to go somewhere, it’s only possible when my husband is at home. Now he works on a 15-day rotational shift. So, only when he comes back, I can go out to take care of things. I cannot go wherever I want. I cannot even enroll my children in clubs or extracurricular activities, because that would mean leaving the house for several hours. And we cannot leave our parents [both require care] unattended. I don’t meet with friends.”

(Informant 1)

“I couldn’t go anywhere, even for groceries I had to leave my mother with someone. Over time, you get used to it and already forget about your personal life…”

(Informant 5)

“It’s not always possible to go when you need to. You depend primarily on her [mother] and also on your sister, because if I’m not staying with her, then I have to ask my sister.”

(Informant 8)

### 3.4. Gender Roles in the Care of Older Adults

Most participants in our study who provided care for older family members were women. Three of them provided care for their parents-in-law, and in some cases caregiving responsibilities were linked to marriage and the role of a daughter-in-law. Five participants cared for their mothers, and one woman cared for her grandmother. Participants’ accounts suggested that caregiving responsibilities were closely intertwined with gendered family expectations when caring for older parents and parents-in-law. Moreover, in several cases, caregiving extended to multiple older adult family members, which was further complicated by the simultaneous need to care for children.

“I have been caring for my mother-in-law for 18 years, ever since I married my husband. She had a stroke shortly after our wedding. My husband is the youngest son in the family, and according to Kazakh tradition, the youngest son is expected to provide care. Whether you want to or not, it’s expected.”

(Informant 2)

“My husband is at work and physically absent for most of the time. We provide care together with my mother, just the two of us. My mother has a disability. We share household chores: I cook, and my mother washes the dishes. If I had time, I would bathe my grandmother; if not, my mother would do it.”

(Informant 10)

Participants’ accounts also highlighted culturally embedded expectations associated with the role of the *kelin* (daughter-in-law). One participant described how her mother continued to view her daughter-in-law primarily through this family role, despite the daughter-in-law’s own age, health, and caregiving responsibilities for her husband:

“Whatever her own situation or health condition, when she comes to my mother, she is still her *kelin* [daughter-in-law]. My mother forgets that she is also caring for her husband.”

(Informant 8)

Gender-related considerations were also evident in one participant’s account of providing intimate personal care. One participant, who had been caring for her mother-in-law, recalled her concerns when her father-in-law was hospitalized following a stroke and she considered the possibility that he might also require her care:

“Because he was a man… I thought he would feel embarrassed if I cared for him.”

(Informant 9)

### 3.5. Insufficient Support from Healthcare and Social Services

This theme captured caregivers’ experiences of limitations in the professional support provided by healthcare and social services. The central issue was not simply the absence of particular services, but the perceived lack of a systematic professional response to caregivers as individuals with health, psychological, and support needs of their own. Caregivers described a lack of understanding and empathy from professionals regarding the challenges they encountered while providing care.

“When we invited the neurologist, she didn’t note any of this, and I did not receive the allowance. She saw my mother-in-law’s health condition—she had suffered three strokes, could not move, had bedsores—but none of this was recorded.”

(Informant 2)

“They [medical staff] did not provide me with any guidance on caregiving. I feel that with a disease like [Alzheimer’s] they don’t treat the person as a person. When her leg swelled, they didn’t even check it. They were dismissive. She is ill, well, so be it.”

(Informant 5)

Together, these experiences illustrate how gaps in professional response extended beyond the care recipient’s clinical needs to the practical consequences of caregiving for the caregiver. Inadequate documentation, limited guidance, and dismissive interactions could leave caregivers without both formal assistance and professional support.

Participants also described a lack of support targeted specifically at them. They were not recognized as a distinct group with their own support needs and generally accessed healthcare and social services only in relation to the care recipient or when they actively requested assistance. Psychological support was not routinely offered, despite participants describing considerable emotional strain.

“I personally asked her [the doctor] for some sedatives, but she did not give or prescribe any—only for the patient. She could see I was nervous, on edge, completely lost. It is very hard to sit at home like in a cage. Even my relatives asked them [the doctors] if they could prescribe something for me, and they said no.”

(Informant 5)

“We don’t receive any support from the clinic. Doctors and nurses only come when called. No one monitors her. We provide her care ourselves. Her daughter is a doctor. She lives in another region, advises us over the phone, and we provide the treatment.”

(Informant 1)

This pattern suggests that formal services were perceived as primarily centered on the care recipient, while caregivers’ own psychological and support needs remained largely outside routine professional attention. Support was therefore described as reactive rather than proactive, with caregivers themselves having to initiate requests for assistance.

Caregivers expressed a desire for recognition and appreciation. For example, daughters caring for their older mothers stated:

“…of course, one wants attention. For instance, if some organization came to us and said, ‘Well done for taking care of your mothers,’ but of course, we don’t have that in Kazakhstan.”

(Informant 3)

“But, if suddenly, for example, someone called me and asked, ‘How are you?’ That would be enough for me. I would know that such an organization exists. There are such people.”

(Informant 8)

Importantly, caregivers understood support not only in terms of tangible services but also as recognition of their caregiving role and their own needs. Even simple expressions of concern were described as meaningful, highlighting their perceived lack of visibility within formal systems of care.

### 3.6. Unmet Caregiver Needs

Caregivers identified several practical and personal needs that they considered important for sustaining their caregiving role while maintaining their own well-being and everyday life. These needs centered primarily on respite, temporary assistance with caregiving, shared caregiving responsibilities, opportunities for rest and personal activities, and employment protection. Most respondents emphasized a need for support from others, particularly short-term assistance that would allow them to rest, take a break, or leave the house for a while, knowing that their relative was in safe hands.

“Perhaps an assistant who could stay with my parents at least sometimes, on a regular basis during the week, would help me so that I could go out and get some distraction.”

(Informant 1)

“I just want to rest, for two or three days, I can put it that way. But not to leave forever, to abandon everything and say: ‘Live however you want.’ No. I don’t even have such thoughts.”

(Informant 3)

“I needed a social worker. And so, when I was refused, I burst into tears right there in the office, because does it mean that people with Alzheimer’s are not considered human? I felt so hurt. I cried. I said, I don’t need someone to clean or cook, I just need someone to watch over [her] for a couple of hours so I could go out and buy groceries.”

(Informant 5)

A daughter caring for her mother explained that if someone else could also help them, they would be able to share the caregiving time, which would allow for more free time.

“If some other family members, for example, happened to be at home, then we could divide those 24 h not between two people, but among more.”

(Informant 8)

Participants described respite as an opportunity to temporarily step away from continuous caregiving demands while remaining committed to their caregiving role. Even short periods of assistance could provide time to attend to personal needs, leave the home, or rest.

Caregiving demands also limited opportunities for caregivers to attend to their own health and personal activities. Lack of time and energy and persistent fatigue were described as barriers to physical activity. This was illustrated by the account of a man caring for his grandmother and uncle:

“I miss my workouts because I need to take care of my grandmother and uncle. Lately, I have been gaining weight.”

(Informant 6)

In addition, respondents expressed concern about keeping their jobs while providing care for older relatives:

“Maybe something like maternity leave, so that the workplace is retained, the years of service continue, but at the same time you are temporarily not working. Some kind of leave to care for a sick or older person.”

(Informant 9)

The need for support also extended beyond immediate caregiving tasks to the preservation of caregivers’ employment and professional roles. The possibility of temporary caregiving leave was viewed as a way to reconcile caregiving responsibilities with continued attachment to employment.

### 3.7. Limited Awareness of Available Support and Services

Distinct from caregivers’ unmet support needs, this theme focused on their awareness of existing health and social services and their ability to obtain information about and navigate available forms of support. Most participants described having to independently search for information on how to perform caregiving procedures and where to seek assistance, relying mainly on the Internet, relatives, or acquaintances. Caregivers were also often unaware of potentially available forms of support, including disability-related social services such as wheelchair-accessible transportation. Information about these opportunities was not routinely communicated to them, leaving caregivers to identify and navigate available support largely on their own.

“I found out about this [allowance and hygiene supplies] from my relatives. They told me that I should go to the social services. Not a single person at the primary healthcare informed us about it. When I went there to inquire, the staff responded very reluctantly, and it was extremely difficult to get them engaged in the process.”

(Informant 2)

“No, nobody provided me with any consultation. I search for all the necessary information on caregiving on the internet, and my husband’s sister advises me on what and how to do. I constantly consult with her.”

(Informant 1)

“ I did not know that we were entitled to use wheelchair-accessible taxi to get to the clinic. I used to take her by regular taxi, but it was difficult for me alone to bring her down and help her into the car, as she was unable to walk.”

(Informant 5)

“No. I watched everything on YouTube about how patients on hemodialysis should eat—what their diet should be.”

(Informant 9)

Participants also reported difficulties in navigating the administrative procedures required to access specialized social services.

“I wanted to obtain legal guardianship and went through the court. After the court decision, I still went to the clinic. They told me: ‘Well, what can you do? You will just have to endure, there is a lot of bureaucracy.’ I eventually stopped going, as no one seemed to care…”

(Informant 10)

Lack of information made it more difficult for caregivers to identify available support and understand how to access it.

## 4. Discussion

This study is one of the first qualitative investigations exploring the caregiving experiences of informal caregivers of older adults in Kazakhstan. Its aim was to provide a deeper understanding of the experiences and circumstances associated with high caregiver burden identified in the preceding quantitative phase. The qualitative findings contextualized the quantitative results by providing further insight into caregivers’ physical and psychological strain and the impact of caregiving on employment, while also expanding understanding of the social and gender-related context of caregiving. In addition, the qualitative phase identified aspects not directly captured by the quantitative measures, particularly limitations in professional support, unmet needs for practical assistance and respite, and limited awareness of available services.

The quantitative phase showed that better caregiver self-rated health was associated with lower odds of high caregiver burden [16], while the interviews provided further context for this association physical exhaustion, sleep disruption, and emotional strain related to their caregiving responsibilities. Similar physical and psychological consequences of caregiving have been reported among informal caregivers of older adults in other settings [28]. Within the Stress Process Model, these direct caregiving demands can be understood as primary stressors [18].

Caregiving responsibilities also affected participants’ employment and their ability to balance work and family roles. In the quantitative phase, approximately one-quarter of caregivers reported changes in their work schedules due to caregiving responsibilities [16]. The qualitative findings provided further context for this finding, as participants described adjusting their working hours, taking time away from work, and, in some cases, leaving employment because of caregiving responsibilities. Similar employment-related consequences have been reported internationally. The AARP reported that more than half of caregivers adjusted their work schedules, 15% reduced their working hours, and 7% declined career advancement opportunities [29], while the S&P Global report documented comparable challenges in balancing caregiving and employment [30]. Participants in our study also described limited understanding from employers and a lack of flexible working arrangements, which made it more difficult to combine employment with caregiving responsibilities [31]. Competing responsibilities extended beyond employment, as some caregivers described tensions between caring for an older relative and fulfilling other family roles, including parenting [32]. Caregiving also restricted participants’ social participation, particularly when they were unable to leave the care recipient without someone to temporarily take over their responsibilities. Reduced opportunities to leave the home and maintain contact with friends contributed to social isolation. Similar restrictions in social interaction and increased social isolation or loneliness have been reported among informal caregivers in other settings [33]. Within the Stress Process Model, these employment, family, and social pressures can be understood as secondary role strains [18].

Participants’ accounts highlighted gendered expectations surrounding caregiving within the Kazakh family context. Women also constituted the majority of caregivers in the preceding quantitative phase [16]. In the qualitative interviews, caregiving responsibilities appeared to be shaped not only by gender but also by culturally embedded expectations regarding family obligations [34]. In particular, daughters and daughters-in-law described assuming substantial responsibility for the everyday care of older relatives. One participant linked her long-term caregiving role as a daughter-in-law to the Kazakh tradition that the youngest son is expected to care for his parents, illustrating how such family expectations may also extend to daughters-in-law. These accounts suggest that gender and family position may intersect in shaping caregiving responsibilities within Kazakh families. Given that nine of the ten participants were women, these findings should be interpreted as reflecting the experiences of this predominantly female sample rather than broader gender differences in caregiving.

Participants’ accounts also highlighted interconnected but distinct challenges related to formal support, unmet needs, and awareness of available services. Insufficient support from healthcare and social services was reflected in limited professional guidance, psychological support, and recognition of caregivers’ role. At the same time, caregivers expressed unmet needs for practical assistance, respite, and support that would enable them to maintain personal and professional roles. A separate issue was limited awareness of available health and social services, with some participants reporting uncertainty about what support was available and where to seek assistance. Although conceptually distinct, these challenges may interact in practice, as limited information can restrict access to existing services, while insufficient professional support may leave caregivers’ needs unmet. Within the Stress Process Model, limited access to information and support may reduce the resources available to caregivers for managing caregiving-related stress [18]. Similar challenges have been documented in other countries, where access to psychological services is limited by stigmatization, mistrust in their effectiveness, and the weak integration of psychological support into primary healthcare [35,36].

From a nursing and primary healthcare perspective, participants’ accounts suggest several areas in which caregiver support could be strengthened. Nurses may be well positioned to identify informal caregivers during routine encounters and conduct brief assessments of caregiver burden and support needs. Depending on identified needs, support could include practical guidance on day-to-day caregiving tasks, information about available health and social services, referrals to psychological support or respite services where available, and follow-up with caregivers experiencing substantial strain. These priorities emerged from participants’ accounts. However, the effectiveness and feasibility of these approaches in the Kazakhstani primary healthcare context require further evaluation.

### Strengths and Limitations

Participants were purposively selected from the preceding quantitative phase based on high caregiver burden (CSI+ ≥ 7). Therefore, the findings primarily reflect the experiences of this group and may have limited transferability to caregivers with lower levels of burden. Most participants were women, limiting the representation of male caregivers’ perspectives and precluding meaningful comparisons by gender. Participants were recruited from urban settings, and 90% had higher education, which may limit transferability to caregivers living in rural areas or those with different educational backgrounds. As data were collected at a single time point, changes in caregivers’ experiences and needs over the caregiving trajectory could not be examined longitudinally, and participants’ descriptions of changes over time should therefore be interpreted as retrospective accounts. Because caregiver burden was not reassessed immediately before the interviews, participants’ level of burden may have changed between the quantitative assessment and the qualitative interview.

Despite these limitations, the study has several strengths. Its sequential explanatory design enabled the qualitative phase to build directly on the preceding quantitative findings and provide a more in-depth understanding of the circumstances underlying high caregiver burden. The use of in-depth semi-structured interviews generated rich accounts of caregivers’ physical, psychological, social, and occupational experiences and their unmet support needs. Recruitment across four geographically distinct cities broadened the range of urban caregiving contexts represented in the study. Furthermore, this study addresses an important evidence gap, as qualitative research on the experiences and support needs of informal caregivers of older adults in Kazakhstan and the broader Central Asian context remains limited. The findings therefore provide context-specific insights that may inform the development of more caregiver-responsive health and social support services.

## 5. Conclusions

This study provides insights into the experiences of informal caregivers of older adults who were purposively selected based on high caregiver burden. Participants described substantial physical, psychological, occupational, and social challenges, as well as gendered expectations surrounding family caregiving and limited professional and social support. They also identified unmet needs for respite, practical guidance, information, and recognition of their caregiving role. These findings highlight the importance of strengthening caregiver support within primary healthcare and improving access to information, practical assistance, and respite services.

## Figures and Tables

**Table 1:** Demographic characteristics of caregivers (N = 10).

ID	Gender	Age (Years)	Marital Status	Education	Employment	Relationship	Number of Care Recipients	Health Problems	Care Experience (Years)
1	F	41	Married	Higher education	Unemployed	Mother-in-law Father-in-law	Two	Dementia or memory impairment and Visual impairment	7
2	F	35	Married	Higher education	Unemployed	Mother-in-law	One	Stroke	18
3	F	58	Single	Higher education	Employed	Mother	One	Stroke	3
4	F	52	Married	Higher education	Unemployed	Mother	One	Dementia or memory impairment	1
5	F	40	Single	Secondary education	Unemployed	Mother	One	Dementia or memory impair	7
6	M	28	Married	Higher education	Employed	Grandmother and uncle	Two	Age-related health problems	10
7	F	35	Single	Higher education	Employed	Mother	One	Visual impairment	6
8	F	56	Single	Higher education	Employed	Mother	One	Age-related health problems	5
9	F	35	Married	Higher education	Employed	Mother-in-law	One	Diabetes mellitus	9
10	F	29	Married	Higher education	Employed	Grandmother	One	Dementia or memory impairment	3
Mean ± SD		40.9± 10.8							

## Data Availability

The qualitative data generated during this study are not publicly available due to participant confidentiality and ethical considerations. De-identified data may be available from the corresponding author upon reasonable request, subject to applicable ethical and confidentiality restrictions.

## Supplementary Materials

The following supporting information can be downloaded at: https://www.mdpi.com/article/10.3390/nursrep16100358/s1, Table S1: Semi-structured interview guide, Table S2: Themes, subthemes, and illustrative codes derived from the reflexive thematic analysis; and the completed Consolidated Criteria for Reporting Qualitative Research (COREQ) checklist.

## Public Involvement Statement

No public involvement in any aspect of this research.

## Guidelines and Standards Statement

This manuscript was reported in accordance with the COREQ for qualitative research.

## Abbreviations

The following abbreviations are used in this manuscript:

WHO	The World Health Organization
ADL	Activities of Daily Living
IADL	Instrumental Activities of Daily Living
COREQ	Consolidated Criteria for Reporting Qualitative Research
