# Supplementary material for: Unmet Needs and Caregiving Burden Among Informal Caregivers of Older Adults: A Qualitative Study
Source: Nurs Rep. 2026 Oct 10;16(10):358. doi: 10.3390/nursrep16100358 (PMC13708490; doi:10.3390/nursrep16100358)
Supplement: Supplementary file 1 [file nursrep-16-00358-s001.zip › nursrep-4559234-supplementary.pdf]

Table S1. Semi-structured interview guide

Main questions	Principal probes
1. Please tell me about your experience of caring for your older family member.	When did you start providing care? How has caregiving changed over time? What types of physical and psychological care do you provide? Has caregiving become more challenging?
2. How has caregiving affected your own life and well-being?	Physical and emotional health? Family life, work, and social activities? Time for yourself?
3. Please tell me about how caregiving responsibilities are shared within your family.	Who is mainly responsible for care? Who else helps? Who helps during difficult periods?
4. What concerns do you have about provided care in the future?	How might care needs change? What plans do you have for future care?
5. What needs have arisen for you as a result of providing care?	What support have you received? What additional practical, emotional, or other support would help you or the older adult?
6. Please tell me about your experiences of obtaining support from healthcare and social care services.	Access to doctors, nurses, or social workers? Advice about caregiving? Follow-up? Support for your own psychological well-being? Where do you obtain information about available services and support for caregivers?
7. What do you think could be done to better support informal caregivers?	What types of assistance are needed? How could support be made more accessible? Is there anything else you would like to add?

**The interview guide was used flexibly. The order and wording of questions varied according to participants' accounts, and probes were used as appropriate to encourage elaboration.*

Table S2. Themes, Subthemes, and Illustrative Codes

Theme	Subtheme	Illustrative codes
Physical and Psychological Impact of Caregiving	Increasing physical demands with greater care dependency	Lifting and transferring; bathing and hygiene; increasing care demands
	Emotional and psychological strain	Sleep disruption; irritability; emotional exhaustion; need for psychological support
Work–Care Balance and Social Isolation	Challenges in balancing employment and caregiving	Leaving employment; adjusting work schedules; taking time off
	Restrictions on social participation and leaving the home	Inability to leave home; reduced contact with friends; dependence on others for replacement
Gender Roles in the Care of Older Adults	Gendered social and cultural expectations of family caregiving	Caregiving by daughters and daughters-in-law; family traditions; gendered expectations
	Perceived personal responsibility for providing care	Feeling that there is no one else to provide care; responsibility for multiple family members
Insufficient Support from Healthcare and Social Services	Lack of guidance and professional support	Lack of caregiving guidance; limited psychological support; difficulties obtaining services
	Lack of recognition, empathy, and attention to caregivers' needs	Feeling ignored by professionals; lack of empathy; caregivers not recognized as a distinct group
Unmet Caregiver Needs	Need for respite and practical caregiving assistance	Need for rest; temporary relief from caregiving; assistance from others; sharing caregiving responsibilities
	Need to maintain personal and professional roles	Time for personal activities; maintaining employment; need for caregiving leave
Limited Awareness of Available Support and Services	Limited information about available support and services	Lack of proactive information; unawareness of available services and benefits; uncertainty about where to seek assistance
	Reliance on self-directed and informal sources of information	Internet and YouTube searches; advice from relatives; medically trained family members; acquaintances with similar experience

COREQ (COnsolidated criteria for REporting Qualitative research) Checklist

A checklist of items that should be included in reports of qualitative research. You must report the page number in your manuscript where you consider each of the items listed in this checklist. If you have not included this information, either revise your manuscript accordingly before submitting or note N/A.

Topic	Item No.	Guide Questions/Description	Reported on Page No.
**Domain 1: Research team and reflexivity**			
Personal characteristics			
Interviewer/facilitator	1	Which author/s conducted the interview or focus group?	3
Credentials	2	What were the researcher's credentials? E.g. PhD, MD	3
Occupation	3	What was their occupation at the time of the study?	3
Gender	4	Was the researcher male or female?	3
Experience and training	5	What experience or training did the researcher have?	3
Relationship with participants			
Relationship established	6	Was a relationship established prior to study commencement?	4
Participant knowledge of the interviewer	7	What did the participants know about the researcher? e.g. personal goals, reasons for doing the research	4
Interviewer characteristics	8	What characteristics were reported about the inter viewer/facilitator? e.g. Bias, assumptions, reasons and interests in the research topic	4
**Domain 2: Study design**			
Theoretical framework			
Methodological orientation and Theory	9	What methodological orientation was stated to underpin the study? e.g. grounded theory, discourse analysis, ethnography, phenomenology, content analysis	4
Participant selection			
Sampling	10	How were participants selected? e.g. purposive, convenience, consecutive, snowball	3
Method of approach	11	How were participants approached? e.g. face-to-face, telephone, mail, email	4
Sample size	12	How many participants were in the study?	4
Non-participation	13	How many people refused to participate or dropped out? Reasons?	4
Setting			
Setting of data collection	14	Where was the data collected? e.g. home, clinic, workplace	4
Presence of non-participants	15	Was anyone else present besides the participants and researchers?	4
Description of sample	16	What are the important characteristics of the sample? e.g. demographic data, date	5-6
Data collection			
Interview guide	17	Were questions, prompts, guides provided by the authors? Was it pilot tested?	4
Repeat interviews	18	Were repeat inter views carried out? If yes, how many?	No
Audio/visual recording	19	Did the research use audio or visual recording to collect the data?	4
Field notes	20	Were field notes made during and/or after the inter view or focus group?	4
Duration	21	What was the duration of the inter views or focus group?	4
Data saturation	22	Was data saturation discussed?	5
Transcripts returned	23	Were transcripts returned to participants for comment and/or	No

Topic	Item No.	Guide Questions/Description	Reported on Page No.
		correction?	
**Domain 3: analysis and findings**			
Data analysis			
Number of data coders	24	How many data coders coded the data?	4
Description of the coding tree	25	Did authors provide a description of the coding tree?	Suppl.Table 2
Derivation of themes	26	Were themes identified in advance or derived from the data?	4-5
Software	27	What software, if applicable, was used to manage the data?	No
Participant checking	28	Did participants provide feedback on the findings?	No
Reporting			
Quotations presented	29	Were participant quotations presented to illustrate the themes/findings? Was each quotation identified? e.g. participant number	6-10
Data and findings consistent	30	Was there consistency between the data presented and the findings?	6-10
Clarity of major themes	31	Were major themes clearly presented in the findings?	6-10
Clarity of minor themes	32	Is there a description of diverse cases or discussion of minor themes?	10-11

Developed from: Tong A, Sainsbury P, Craig J. Consolidated criteria for reporting qualitative research (COREQ): a 32-item checklist for interviews and focus groups. *International Journal for Quality in Health Care*. 2007. Volume 19, Number 6: pp. 349 – 357

Once you have completed this checklist, please save a copy and upload it as part of your submission. DO NOT include this checklist as part of the main manuscript document. It must be uploaded as a separate file.
